# Animal-Assisted Interventions: Factors Affecting Donkey Behaviours and Attitude Toward Humans

**DOI:** 10.3390/ani14213139

**Published:** 2024-11-01

**Authors:** Serenella d’Ingeo, Valeria Straziota, Marcello Siniscalchi, Onofrio Depalma, Sara Petrassi, Michela Romano, Angelo Quaranta

**Affiliations:** 1 Animal Physiology and Behaviour Research Unit, Department of Veterinary Medicine, University of Bari Aldo Moro, 70121 Bari, Italy; valeria.straziota@uniba.it (V.S.); marcello.siniscalchi@uniba.it (M.S.); angelo.quaranta@uniba.it (A.Q.); 2 Società Cooperativa Sociale Comunità Oasi2 San Francesco Onlus, 76125 Trani, Italy; o.depalma@oasi2.it; 3 Freelance Psychologist, 70121 Bari, Italy; sepetrassi@gmail.com; 4 GEA ETS Centro Studi Interventi Assistiti con gli Animali, 36015 Schio, Italy; michelaromano@gea.pet

**Keywords:** donkeys, animal-assisted interventions, welfare, behaviour, migrants

## Abstract

The relationship between humans and donkeys has ancient origins. In recent years, donkeys’ involvement in Animal-Assisted Intervention (AAI) has grown, raising major concerns about their welfare during these activities. We investigated the factors affecting donkeys’ behaviour during an AAI programme for migrants. We observed an effect of human distance from the animal, the interaction mode, and food presence on the animals’ behaviours and attitudes toward humans. Moreover, we provide preliminary evidence of the effect of AAI on migrants’ welfare and prosocial behaviours and offer insights into the issues to address in future studies.

## Simple Summary

Donkeys are frequently involved in activities with humans, which include educational and recreational activities and therapy (i.e., Animal-Assisted Intervention, AAI). To date, little is known about the factors that could affect animal welfare during AAI. Moreover, no studies have investigated the potential benefit of Donkey-Assisted Intervention on the welfare of migrants hosted by public social services. We observed that human distance from the animal, the interaction mode, and food presence significantly impacted the animal’s behaviours and attitudes toward humans. Furthermore, we provide evidence of the effect of AAI on migrants’ welfare.

## 1. Introduction

The relationship between humans and donkeys has ancient origins that can be traced back to approximately 5000 years ago [1]. It was mainly based on the support of donkeys in human activities and work, which helped promote the economic and technological development of human society [2,3,4]. More recently, donkeys have found a different role as companion animals and are frequently involved in Animal-Assisted Intervention (AAI) [5]. AAI is a general term indicating the “goal oriented and structured interventions” [6] that use animals “to improve the physical, mental, and social conditions of humans” [3]. Specifically, a growing body of literature shows that Donkey-Assisted Interventions (DAIs) enhance human mental health, particularly in the management of emotions and communication abilities [7,8]. Human interaction with donkeys efficiently supports the rehabilitation process of adults with intellectual disability [9] and children with dyslexia by improving self-esteem and school performance [10]. However, the use of animals in AAI has raised major concerns for their welfare in both the general population and the scientific community [3]. The effectiveness of AAI is, indeed, based on the One Health–One Welfare principle, which states that human health and wellness is strongly related to animal health and welfare and a positive human–animal relationship [11]. Despite the widespread use of donkeys in AAI, the current literature on the subject is strikingly scarce and mainly focused on human outcomes [2,11,12]. To date, only two studies have focused on donkeys, particularly on the protocols for the selection of the animals for AAI [2,7]. Specifically, Panzera and colleagues [7] used the “AWIN welfare assessment protocol for donkeys” [13] to assess animal health, together with behavioural tests (i.e., avoidance test, novel object, and unknown person test) to evaluate animals’ suitability for AAI. On the other hand, Gonzales-De Cara and colleagues [2] developed a novel protocol to assess donkey temperament by evaluating the animal’s reactions to sensory (tactile and sound) and social stimuli (reactivity to human) as well as the display of fear-related behaviour toward unknown objects and events. Although the appropriate selection of animals is crucial, a more in-depth evaluation of the factors that could elicit stress or positive behaviours toward humans is necessary for the welfare of the animals involved. According to the Terrestrial Animal Health Code of the World Organisation for Animal Health (OIE) [14], “an animal is in a good state of welfare if (as indicated by scientific evidence) it is healthy, comfortable, well nourished, safe, able to express innate behaviour, and if it is not suffering from unpleasant states such as pain, fear, and distress. […] Animal welfare refers to the state of the animal […]”. Such a definition highlights the need to take the animals’ emotions into account when evaluating their welfare state. Broadly, emotions are defined as short-term affective states (closely linked to internal brain action states) elicited by internal and/or external events which elicit a physiological, behavioural, and cognitive response [15,16,17]. Therefore, the changes produced in animals’ behaviour, physiology, and cognition could be measured to derive information on the individuals’ affective state [18,19]. In the equine literature, several studies have been carried out assessing horses’ experiences of interactions with humans (for a review, see [20]). Emotions, indeed, influence individual social motivation by determining whether the animal approaches or avoids a stimulus or situation [21]. Approach behaviours are generally linked to the positive appraisal of stimuli and are indicative of the expectations of positive outcomes, whilst avoidance behaviours orient the animal away from aversive stimuli and from the threat of negative consequences [22].

To date, evidence of donkeys’ experiences of interactions with humans, including those occurring during AAI, is lacking. This knowledge would allow a better understanding of the potential factors that could impact animal welfare when interacting with humans and could help develop more complex protocols for the correct management of the sessions (e.g., duration or refreshment time) according to each animal’s needs.

Therefore, the aim of this study was to explore the effects of different factors (i.e., human distance from the animals, the type of interaction, and food presence in the AAI set-up) on the behaviour of the donkeys involved in AAI with migrants. Moreover, we investigated, for the first time, the potential benefit of DAI on the welfare of migrants hosted by public social services.

## 2. Materials and Methods

### 2.1. Participants

#### 2.1.1. Donkeys

Four donkeys were involved in the study. They were all females of different breeds (Ragusano, Amiata, Irish, and mixed-breed), aged between 3 and 16 years (9.25 ± 5.38; mean ± S.D.). The animals belonged to three different facilities with similar housing conditions. The donkeys lived in outdoor enclosures with available coverage or stalls and were fed hay, while water was provided ad libitum. Three donkeys lived with another conspecific (two belonged to the same facility), whereas the other lived in a mixed-species group with two ponies and a goat.

All the animals were evaluated by a veterinarian according to the AWIN welfare assessment protocol [13] (i.e., body condition score, absence of injuries and disease, and dehydration) to certify their health. They were all involved in occasional activities with unfamiliar humans with similar frequency (mean of 3 sessions per month) and had similar experience in AAI.

#### 2.1.2. Humans

Twenty-three migrants voluntarily participated in the study. They were divided into two groups: the experimental group (EG), who took part in DAI, and the control group (CG), who was involved in activities related to nature. The EG was made up of 14 men and 1 woman, aged between 16 and 46 years (29.8 ± 9.32; mean ± S.D.); the CG was composed of 8 men, aged between 17 and 36 years (26.12 ± 7.02; mean ± S.D.).

The activities were carried out in the same facilities for both the EG and CG. In particular, the DAI activities were grooming, lunging, and offering food to the animals, whereas the CG activities were gardening, plant recognition, and soil preparation for planting.

### 2.2. Procedure

**Figure 1:**
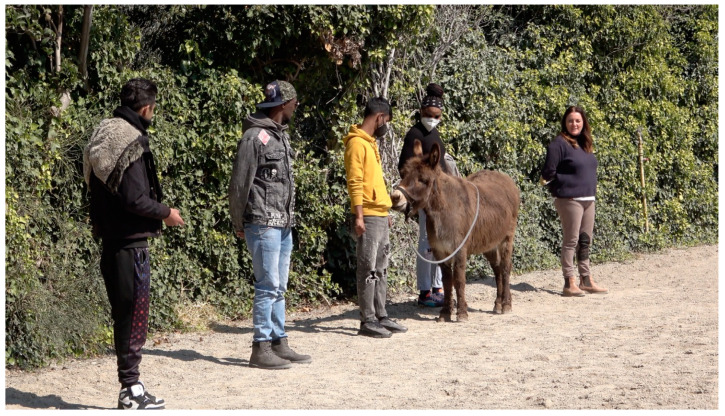
An interaction between humans and one of the donkeys involved in the project.

The activities were carried out weekly over a three-month period. The three different locations hosted the activities on a rotating basis so that each of them (and the host animals) was not visited for two weeks consecutively. Each session (both for the EG and the CG) lasted approximately 40 min. For both the EG and the CG, the activities were supervised and modulated by a psychologist (and the animal assistant for the EG) according to the Italian National Guidelines for Animal-Assisted Interventions [23]. Human–animal interactions (EG) were carried out in the animals’ living environment, in a familiar fenced area (Figure 1).

To evaluate the animals’ affective states and their changes related to their interaction with humans, the donkeys’ behaviour was recorded continuously during the EG activities by two high-resolution cameras (Sony 4K FDR-AX43^®^, Sony, Japan) held by two experimenters. On the other hand, the effects of AAI on the migrants’ welfare were measured using the Refugee Health Screener-15 (RHS-15) [24] and the Prosocial Behavioral Intentions Scale [25]. The RHS-15 is a screener for emotional distress in refugees. It was developed for refugees, but previous studies have used it to analyse stress levels in migrant populations [26]. It is composed of 15 items: items 1–14 are related to the presence of physical and emotional symptoms (e.g., “Feeling restless, can’t sit still” and “Feeling down, sad, or blue most of the time”) and are rated on a 5-point Likert scale ranging from 0 (“not at all”) to 4 (“extremely”); item 15 is a distress thermometer ranging from 0 (no distress: “Things are good”) to 10 (extreme distress: “I feel as bad as I ever have”). The Prosocial Behavioral Intentions Scale is made up of four items rating the willingness to help others. Each item was rated on a 7-point scale ranging from 1 (definitely would not do this) to 7 (definitely would do this). The items included behaviours such as “Comfort someone I know after they experience a hardship”.

The questionnaires were administered to the participants in their native language or a foreign language (English or Italian) according to each person’s preference. The languages used were Arabic, Urdu, Bengali, French, Italian, and English. The questionnaires were completed individually by each participant before the beginning of the project and at its end.

### 2.3. Data Analysis

#### 2.3.1. Donkeys

The video recordings of the activities with the animals were analysed by two trained observers. The analysis of the donkeys’ behaviour was performed using The Observer XT (Noldus^®^, Wageningen, Netherland). The ethogram used was developed to evaluate the animals’ attitudes toward humans following the procedure described by Lerch and colleagues [12]. Specifically, the behaviours were divided into four categories: positive, uncertain, and negative attitudes toward humans, and stress-related behaviour. All the behaviours considered in each category are listed in Table S1 (Supplementary Information Table S1) [12,27,28,29,30]. The frequency of each behaviour considered in the ethogram was analysed, and the total number of behaviours displayed by each donkey for each behavioural category (i.e., positive, uncertain, and negative attitudes toward humans, and stress-related behaviour) was computed. Moreover, human distance from the animals (i.e., 0–1 m, 2–3 m, and >3 m, adapted from [2,28]), the interaction mode (human staying still, human approaching, and physical contact) and food presence in the activity set-up (available food, food offered by humans, and no food available) were evaluated continuously to assess their influence on the animals’ behaviour toward humans.

#### 2.3.2. Migrants

The total score of items 1–14 of the Refugee Health Screener-15 (RHS-15) and the Prosocial Behavioral Intentions Scale was computed. The score of item 15 of the RHS-15 was analysed separately as a measure of distress levels. The scores obtained before and after the activities for both the EG and CG were then analysed to detect differences between the conditions.

### 2.4. Statistical Analysis

The generalised linear mixed-effects model (GLMM) was used to study the effects of the factors examined, i.e., human distance from the animals, the interaction mode, and food presence, on the behavioural categories (positive, uncertain, or negative attitudes toward humans). Data distribution was tested using the Shapiro–Wilk test. Since the values of the tested variables were distributed along a positive scale that was skewed toward larger positive values, the inverse Gaussian distribution and log-link function were used. The Bayesian information criterion (BIC) was employed for selecting and comparing models based on the −2 log likelihood. To detect differences between different groups, Fisher’s Least Significant Difference (LSD) pairwise comparisons were performed.

Regarding the effects of DAI on the migrants’ welfare and prosocial behaviour, Wilcoxon signed rank tests and paired-samples *t*-tests (according to data distribution) were used to test for differences between the scores of the RHS-15 and Prosocial Behavioral Intentions Scales before the beginning and at the end of the project.

## 3. Results

### 3.1. Donkeys

**Figure 2:**
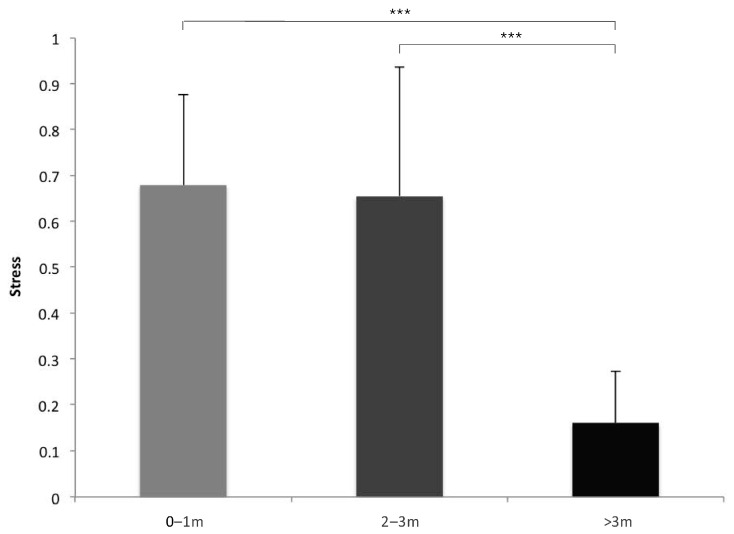
Data of the score of the stress-related behaviour category for each of the distances between the humans and animals considered (means with SEM are shown). ***: *p* < 0.001.

The analysis revealed a significant effect of the human interaction modes on the stress-related behaviour category (F(2,26) = 9,996,121, *p* = 0.000). The post hoc analyses showed that the donkeys displayed more stress-related behaviours in the “still” and “physical contact” interactions than in the “approaching” condition (“still” vs. “approaching”: *p* = 0.000; “physical contact” vs. “approaching”: *p* = 0.000). No difference between “still” and “physical contact” was revealed (*p* = 0.986). A significant effect of the distance between the humans and animals on the stress-related behaviour category was observed (F(2,26) = 1392, *p* = 0.000; Figure 2). Specifically, the analysis showed that the donkeys’ stress level was significantly higher at “0–1 m” and “2–3 m” distances than “>3 m” (“0–1 m” vs. “>3 m”: *p* = 0.000; “2–3 m” vs. “>3 m”: *p* = 0.000); whereas no differences were observed between “0–1 m” and “2–3 m”. No effect of food presence was found (*p* = 0.343).

Considering the animal’s positive attitude toward humans, a significant effect of the human interaction modes was observed (F(6,46) = 438,644, *p* = 0.000). The post hoc analyses showed that the donkeys displayed more positive behaviours toward humans in the “approaching” and “still” conditions than during “physical contact” (“approaching” vs. “physical contact”: *p* = 0.000; “still” vs. “physical contact”: *p* = 0.000), whereas no differences between “still” and “approaching” were found (*p* = 0.195). An effect of food presence on the animals’ positive attitude toward humans was observed (F(6,46) = 555,574, *p* = 0.000). More positive behaviours were displayed in the presence of food (“available food” and “offered food”) than in its absence (*p* = 0.000).

Moreover, significant differences were also found between “available food” and “offered food”: specifically, positive behaviours toward humans were higher when the food was available than when it was offered directly by humans (*p* = 0.000). An effect of the distance between the humans and animals on the positive behaviours was also shown (F(2,26) = 11,906, *p* = 0.000). In particular, positive behaviours were higher at “0–1 m” and “2–3 m” distances than “>3 m” (“0–1 m” vs. “>3 m”: *p* = 0.000; “2–3 m” vs. “>3 m”: *p* = 0.000) and at “2–3 m” than “0–1 m” (*p* = 0.047).

No effect of human interaction modes, food presence, or human–animal distance on the animals’ negative attitude toward humans was found (*p* > 0.05). On the contrary, the analysis showed an effect of food presence (F(2,37) = 7477, *p* = 0.002) and the human–animal distance (F(2,37) = 5476, *p* = 0.007) on the animals’ uncertain attitude toward humans. Specifically, we observed that the uncertainty-related behaviours were higher when no food was available than in the presence of food, both when it was freely available (*p* = 0.006) and directly offered by humans (*p* = 0.013). Moreover, the animals’ uncertain attitude was higher when food was offered by humans than freely available (*p* = 0.015). The post hoc analysis revealed also that uncertainty-related behaviours were higher at a “0–1 m” distance than both at “2–3 m” and “<3 m” distances (“0–1 m” vs. “2–3 m”: *p* = 0.018; “0–1 m” vs. “>3 m”: *p* = 0.007). No differences were found between “2–3 m” and “<3 m” distances (*p* = 0.079). No effect of human interaction modes on the animals’ uncertain attitude toward humans was observed (*p* > 0.05).

### 3.2. Migrants

The results of items 1–14 of the Refugee Health Screener scale (RHS-15) showed no differences between the scores obtained before and after the Animal-Assisted Intervention programme for both the control and the experimental group (*p* > 0.05; *t*-test paired samples). Similarly, we observed no differences between the stress scales (item 15 of the RHS-15) measured before and after the end of the activities (*p* > 0.05; Wilcoxon signed rank tests).

The results of the Prosocial Behavioral Intentions Scale showed significant differences in the score measured before and after the activities in the control group, which had a lower score in the post-test phase than in the pre-test (Z = 0.00, *p* = 0.012; Wilcoxon signed rank tests). No significant differences, but a tendency between the pre- and post-test scores, were observed for the experimental group (*p* = 0.057; Wilcoxon signed rank tests), which obtained a higher score in the post-test phase than in the pre-test.

## 4. Discussion

Our preliminary study shows a general effect of the characteristics of human–animal interactions on the animals’ behaviours and attitudes toward humans. Specifically, we observed an effect of human distance from the animal, the interaction mode, and food presence on the animals’ behaviours. Interestingly, the distance between humans and animals appears to play an important role in determining donkeys’ stress levels, which significantly decreased at a distance greater than 3 m. This finding suggests that 3 m may constitute the limit of the animals’ flight zone. The flight zone is defined as the distance at which an animal allows a human (or another individual) proximity without showing signs of stress (e.g., avoidance or moving away) [31]. Previous studies on livestock show that it can vary according to individual factors, including the animals’ wildness or tameness [31]. In equids, it has been shown that the flight zone depends also on the individual’s sex and species: male horses and mares exhibit higher flight zone behaviours (i.e., increasing or maintaining distance away from the approaching equid) than male and female mules when interacting with a conspecific [32]. In free-roaming ponies, the flight zone was reported to vary mainly between three and nine metres [33]. To date, evidence on donkeys’ flight zones is lacking. However, tests evaluating animals’ responses to humans’ approach have been developed [2,13]. Specifically, in the European Animal Welfare Indicators (AWIN) protocol for donkeys, Minero and colleagues [13] included the “avoidance distance” test to assess the quality of the human–animal relationship. The animals’ avoidance response (i.e., moving away or turning their head away) is registered by an observer who stands in front of the animals at around 3.5 m raising an arm at 45° from their chest, and walks calmly and slowly toward the animals. Similarly, Gonzales-De Cara and colleagues [2] evaluated the animals’ reactivity to humans through the unfamiliar passive human test (UPH), in which a person stands motionless in the testing area, and the unfamiliar active human test (UAH), where a person moves slowly from 6 m toward the donkey, trying to touch its back and muzzle. Both the UPH and the UAH, together with tests evaluating reactivity to sounds, novel objects, and tactile stimulation, have been proposed to select animals for assisted therapy [2]. Our results are in line with the initial distance considered in the UAH test, suggesting that distances greater than 3 m are needed for a proper evaluation of the animal’s emotion-related perception of humans. In this respect, the factors influencing an individual’s flight zone deserve further investigation in order to define an appropriate starting distance for evaluating the animal’s response to human presence and approach.

We observed that at closer distances (i.e., 0–1 m and 2–3 m), the donkeys’ attitudes toward humans significantly varied: a higher frequency of positive behaviours at medium distance (2–3 m) was registered, whereas at a very close distance (0–1 m), the donkeys displayed both positive and uncertain attitudes toward the humans. This result suggests that additional factors could contribute to determining the animals’ attitudes toward humans during their interactions beyond distance alone. Our results indicate that food could play a major role in promoting positive behaviour. Indeed, we found a higher frequency of positive behaviour when food was present in the AAI set-up, particularly when it was freely available. On the contrary, we observed more behaviours related to an uncertain attitude in the absence of food. Regarding the food directly offered by humans, our results suggest that might have had an ambiguous value for the animals as it elicited behaviours related to both positive and uncertain attitudes. The use of food directly offered to the animals during Animal-Assisted Intervention could promote the animals’ willingness to approach humans, which is generally required for the activities. However, the approaching behaviour might not be a clear expression of an animal’s willingness to interact with humans, but rather it could be only related to an animal’s motivation to obtain food. Specifically, a highly food-motivated animal could approach humans to obtain food even if it feels uncomfortable or mildly stressed by the human’s presence. This would undermine the efficacy of the intervention, which is based on the reciprocity of the interactions and the experience of positive emotions for both the humans and the animals [34]. Therefore, the use of food to mediate the interaction with an animal is worthy of special consideration, both for the selection of the animals and during the activities. The selection of animals for Animal-Assisted Intervention needs to be carefully calibrated according to the subject’s real attitude to interactions with humans without the mediation of food. This assessment would also be useful to shape the type of activities in which the animal will be participating (e.g., talk therapy, training, or physical activities) [34].

As for the interaction mode, the results provide interesting information about the effect of physical contact on the animals’ experience of AAI. We observed, indeed, that the interactions with humans involving tactile stimulation increased the animals’ stress levels (i.e., more stress-related behaviours) and elicited fewer positive behaviours toward humans. This evidence offers important insight into the animals’ experiences of tactile interactions, which are commonly present in AAI practice, and raises key concerns for the animals’ welfare. Although animals are expected to tolerate physical contact with strangers during AAI, there is clear evidence that physical interactions may be perceived as unpleasant or could even elicit discomfort and conflicts [35]. On the human side, although a beneficial effect of physical contact on stress reduction has been reported (i.e., it reduces salivary cortisol levels [36]), appropriate control conditions are lacking, making the effectiveness of body contact unclear [37]. Specifically, it is debatable whether the physical contact with the animal or the presence of the animal per se provides the benefit of buffering against stress [37]. Overall, there are several issues related to the benefits of physical contact during AAI for both the humans and the animals involved that deserve deeper investigation.

Our results shed light on the need to deepen the knowledge of the effects on human distance from the animal, the interaction mode, and food presence (and their interaction) on the animals’ behaviours during AAI. Future studies examining a larger sample are necessary to support our results and hypothesis. Moreover, further investigation into both the behavioural and physiological response (e.g., heart rate variation, cortisol level, and brain activity [18,38,39]) to humans is required to provide more complex results on the animal’s emotional functioning during the interaction with humans along the valence dimension (positive or negative) and arousal level [40]. Our results showed no significant effect of AAI on the welfare of the participating migrants. Specifically, we found no differences in the scores of general mental health, stress levels (measured by the Refugee Health Screener [24]), or prosocial behaviours (measured by the Prosocial Behavioral Intentions Scale [25]) before and after the AAI. Similarly, no differences in the general mental health and stress levels were observed for the control group which took part in nature-based activities. These results suggest that both AAI and nature-based activities might not impact migrants’ welfare in the long term. The activities, indeed, were carried out over a period of three months (with a weekly interval), during which other major causes of severe stress, including job search, bureaucratic procedures for citizenship, and cohabitation with unknown individuals in a different country, could have contributed to mental health conditions. These factors could have mitigated the potential benefits of the activities. In our study, the plant-nurturing activity was selected for the control group to account for any effect of the change in environment and the practical activity involving a group of different people on stress levels and prosocial behaviours. However, future studies are needed to evaluate if the presence of the animals is sufficient for promoting socialisation and reducing stress with respect to the interaction with the animals.

Our study is the first to examine the benefits of the interaction with animals on migrants’ well-being, highlighting the need to increase knowledge on this topic in order to mitigate stress, improve mental health [41], and promote integration into Italian society. Previous studies reported, indeed, that creative art therapy [42] and social farming [43] successfully support individuals in the adaptation to a new environment. Therefore, future studies are needed to shed light on the external factors that could impact individual mental health in the long term and the beneficial effects of AAI (both clinical and psychoeducational). For instance, the use of tools for measuring the effects of the interventions by comparing the human stress levels before and after each session could offer more accurate information about the effects of AAI in the short term.

## 5. Conclusions

Our results provide the first evidence of donkeys’ experiences during AAI, highlighting the influence of different factors that could impact the animals’ emotional state during the activities. Further investigation of the relationship between such factors and their effect on the animals’ emotional perception of the interaction with humans is needed to identify accurate parameters to assess animal welfare during AAI. Moreover, we provide evidence of the effect of AAI on migrants’ welfare and prosocial behaviours, highlighting the potential limits of the methodology used to investigate these aspects. Finally, we offer insight into the critical issues that need to be considered in future studies for exploring migrants’ welfare state and the benefit of human–animal interactions for participating migrants.

## Data Availability

The datasets generated and/or analysed during the current study are available from the corresponding author upon reasonable request.

## Supplementary Materials

The following supporting information can be downloaded at https://www.mdpi.com/article/10.3390/ani14213139/s1. Table S1: List of the behavioural categories and the related behaviours scored for the analysis.
