# Supplementary material for: Animal-Assisted Interventions: Factors Affecting Donkey Behaviours and Attitude Toward Humans
Source: Animals (Basel). 2024 Nov 1;14(21):3139. doi: 10.3390/ani14213139 (PMC11545277; doi:10.3390/ani14213139)
Supplement: Supplementary file 1 [file animals-14-03139-s001.zip › animals-3219991-supplementary.pdf]

Animal Assisted Interventions: factors affecting donkeys' emotional state during the interactions with humans

Serenella d'Ingeo[1,*], Valeria Straziota[1], Marcello Siniscalchi[1], Onofrio Depalma[2], Sara Petrassi[3], Michela Romano[4] and Angelo Quaranta[1]

1 Animal Physiology and Behaviour Research Unit, Department of Veterinary Medicine, University of Bari Aldo Moro, 70121 Bari, Italy
2 Società Cooperativa Sociale Comunità Oasi2 San Francesco Onlus, 76125 Trani, Italy
3 Freelance Psychologist, 70121 Bari, Italy
4 GEA ETS Centro Studi Interventi Assistiti con gli Animali, 36015 Schio, Italy

* Correspondence: serenella.dingeo@uniba.it

Table S1. List of the behavioural categories and the related behaviours scored for the analysis [12,27-30].

Behavioural categories	Behaviours
Positive attitude toward humans (ears forward)	Gaze<br>Approach<br>Sniff<br>Lick<br>Nibbling
Negative attitude toward humans (ears backward)	Gaze<br>Approach<br>Sniff<br>Lick<br>Nibbling<br>Threatening bite<br>Threatening approach<br>Threatening kick<br>Kicking<br>Bite clothes<br>Jostling<br>Bite<br>Tucked tail<br>Move away<br>Fly away<br>Refuse to move
Uncertain attitude toward humans (ears asymmetric)	Gaze<br>Approach<br>Sniff<br>Lick<br>Nibbling<br>Move away<br>Fly away<br>Refuse to move
Stress	Pull the rope<br>Stomping<br>Pawing<br>Rubbing<br>Noddling<br>Shanking<br>Tossing<br>Raised/high<br>Head down<br>Teeth grinding<br>Chomping<br>Gaping<br>Yawning<br>Tail swishing
